# Relocation of bioclimatic suitability of Portuguese grapevine varieties under climate change scenarios

**DOI:** 10.3389/fpls.2023.974020

**Published:** 2023-02-08

**Authors:** Filipe Adão, João C. Campos, João A. Santos, Aureliano C. Malheiro, Hélder Fraga

**Affiliations:** ^1^ Centre for the Research and Technology of Agro-Environmental and Biological Sciences (CITAB), University of Trás-os-Montes e Alto Douro (UTAD), Vila Real, Portugal; ^2^ Centre for Research in Geospace Science (CICGE), University of Porto, Vila Nova de Gaia, Portugal

**Keywords:** viniculture, future climate, ecological niche models, ensemble modeling, grape varieties, Europe, BIOMOD 2

## Abstract

**Introduction:**

Climate change has been driving warming trends and changes in precipitation patterns and regimes throughout Europe. Future projections indicate a continuation of these trends in the next decades. This situation is challenging the sustainability of viniculture and, thus, significant efforts towards adaptation should be then carried out by local winegrowers.

**Method:**

Ecological Niche Models were built, using the ensemble modelling approach, to estimate the bioclimatic suitability of four main wine-producing European countries, namely France, Italy, Portugal, and Spain, in the recent past (1989–2005), for the cultivation of twelve Portuguese grape varieties. The models were then used to project the bioclimatic suitability to two future periods (2021– 2050 and 2051–2080) to better understand the potential shifts related to climate change (modeled after Intergovernmental Panel on Climate Change’s Representative Concentration Pathways 4.5 and 8.5 scenarios). The models were obtained with the modeling platform BIOMOD2, using four bioclimatic indices, namely the “Huglin Index”, the “Cool Night index”, the “Growing Season Precipitation index”, and the “Temperature Range during Ripening index” as predictor variables, as well as the current locations of the chosen grape varieties in Portugal.

**Results:**

All models performed with high statistical accuracy (AUC > 0.9) and were able to discriminate several suitable bioclimatic areas for the different grape varieties, in and around where they are currently located but also in other parts of the study area. The distribution of the bioclimatic suitability changed, however, when looking at future projections. For both climatic scenarios, projected bioclimatic suitability suffered a considerable shift to the north of Spain and France. In some cases, bioclimatic suitability also moved towards areas of higher elevation. Portugal and Italy barely retained any of the initially projected varietal areas. These shifts were mainly due to the overall rise in thermal accumulation and lower accumulated precipitation in the southern regions projected for the future.

**Conclusion:**

Ensemble models of Ecological Niche Models were shown to be valid tools for winegrowers who want to adapt to a changing climate. The long-term sustainability of viniculture in southern Europe will most likely have to go through a process of mitigation of the effects of increasing temperatures and decreasing precipitation.

## 1 Introduction

The vinicultural socio-economic sector is very important in Europe. It employs thousands of people and generates important revenues, which can help support local economies and boost other sectors such as wine tourism (Jenster and Jenster, 1993; Santos et al., 2020; Comité Européen des Entreprises Vins – CEEV, 2022). Furthermore, viniculture can represent an important tool for the sustainability of ecosystems in and around vinicultural regions by diversifying ecological infrastructures and ground cover with different types of plants and weeds (Gomes et al., 2021). Climatic conditions have been favorable for grape production ever since it first started in the continent, mainly in southern Europe, with temperatures and precipitation allowing for proper phenological development of a wide range of grapevine varieties (Santos et al., 2020). However, in the last decades, climatic conditions have been sharply changing, with an overall rise in temperatures and modifications in the precipitation spatial patterns and temporal regimes (IPCC, 2021). Studies have shown that these changes in climatic conditions have already impacted grapevine yield and wine quality in the past (Kenny and Harrison, 1992; Jones et al., 2005), while future projections indicate a strengthening of the previous climatic trends (van Leeuwen et al., 2019; Martins et al., 2021), as well as a higher frequency of extreme weather events, such as heatwaves, droughts, and floods (Fraga et al., 2020; IPCC, 2021). Therefore, it is highly likely that the vinicultural sector will continue to suffer impacts resulting from the alterations in the climate. As such, adaptation measures will need to be taken to ensure its long-term viability (Malheiro et al., 2010; Fraga and Santos, 2018; Santos et al., 2019).

The detrimental impacts of climate change on plants and crops around the world have been discussed for some time now (Easterling et al., 2007; Luedeling et al., 2011). The global rise of temperatures along with reduced precipitation and soil water content, in some regions, point towards an increased pressure on food production, as gradually less landmass will be suitable for agriculture (IPCC, 2021). Regarding viniculture, studies based on bioclimatic indices suited to the analysis of this activity have stated that under the Intergovernmental Panel on Climate Change Representative Concentration Pathways (RCP) 4.5 and 8.5 scenarios, higher heat accumulation and less chilling hours may compromise the long-term sustainability of several vinicultural regions in southern and central Europe (Luedeling et al., 2011; Fraga and Santos, 2021). These regions include, for example, the Alentejo and Douro regions in Portugal, the Andalucía and La Mancha regions in Spain, Campania, Puglia, and Sicily regions in Italy, and the Alsace region in France. Increasingly hotter conditions result in drier terrains, which, in turn, result in a considerable drop in vineyard yields (Duchêne and Schneider, 2005; Kizildeniz et al., 2015; van Leeuwen and Darriet, 2016; Bonfante et al., 2018; van Leeuwen et al., 2019). Conversely, vinicultural regions in central and northern Europe may benefit from a warming climate. Countries like Austria or Hungary are expected to have longer growing seasons and frost-free periods that will allow for greater vineyard yield and better-quality wine (Koundouras et al., 1999; Stock et al., 2005; Bertin, 2008; Neethling et al., 2012; Droulia and Charalampopoulos, 2021; Kovács and Jakab, 2021), though some uncertainties remain due to the complex interplay of factors involving climate change and its impact on viniculture in Europe (Santos et al., 2019).

Ecological Niche Models (ENMs) are powerful tools that may allow for further improvement in the knowledge of this matter. These models are built using correlative computer algorithms, which use the current locations of the species being studied and the associated environmental variables (e.g., topographical, climatic, or landscape variables) (Guisan and Zimmermann, 2000), and estimate wherein a certain study area there are similar climatic conditions to those associated with the species being modeled (Araújo and Peterson, 2012). The application of these models has been done previously to estimate the bioclimatic suitability of Europe for several grape varieties, and its potential change with climate change (Moriondo et al., 2013), using the Random Forests method (Breiman, 2001). More recently, a study focused merely on modeling the Spanish Castille-and-León vinicultural regions’ bioclimatic suitability for several Spanish grape varieties (del Río et al., 2021), considering recent past climatic conditions, using the Maxent method (Phillips et al., 2006). Building on these previous works, ENMs were used to study the bioclimatic suitability of four main wine-producing European countries for twelve main Portuguese grape varieties and their potential change with climate change. Climatic conditions were considered for both the recent past (1989–2005) and the future (2021–2050 and 2051–2080), considering the RCP 4.5 and 8.5 radiative forcing scenarios, and were characterized by four bioclimatic indices, namely the “Cool Night index” (Tonietto and Carbonneau, 2004), the “Huglin Index” (Huglin, 1978), the “Temperature Range during Vine Ripening index” (Mullins et al., 1992), and the “Growing Season Precipitation index” (Blanco-Ward et al., 2007)]. Furthermore, ensemble modeling (Araújo and New, 2007) was implemented to consider model results from different modeling methods. This approach constitutes a novelty concerning the previous studies and, thus, the study offers new and improved insights to the scientific community as well as to winegrowers who want to adapt to a changing climate and ensure the sustainability of their activities in the long term.

## 2 Material and methods

### 2.1 Study area

**Figure 1:**
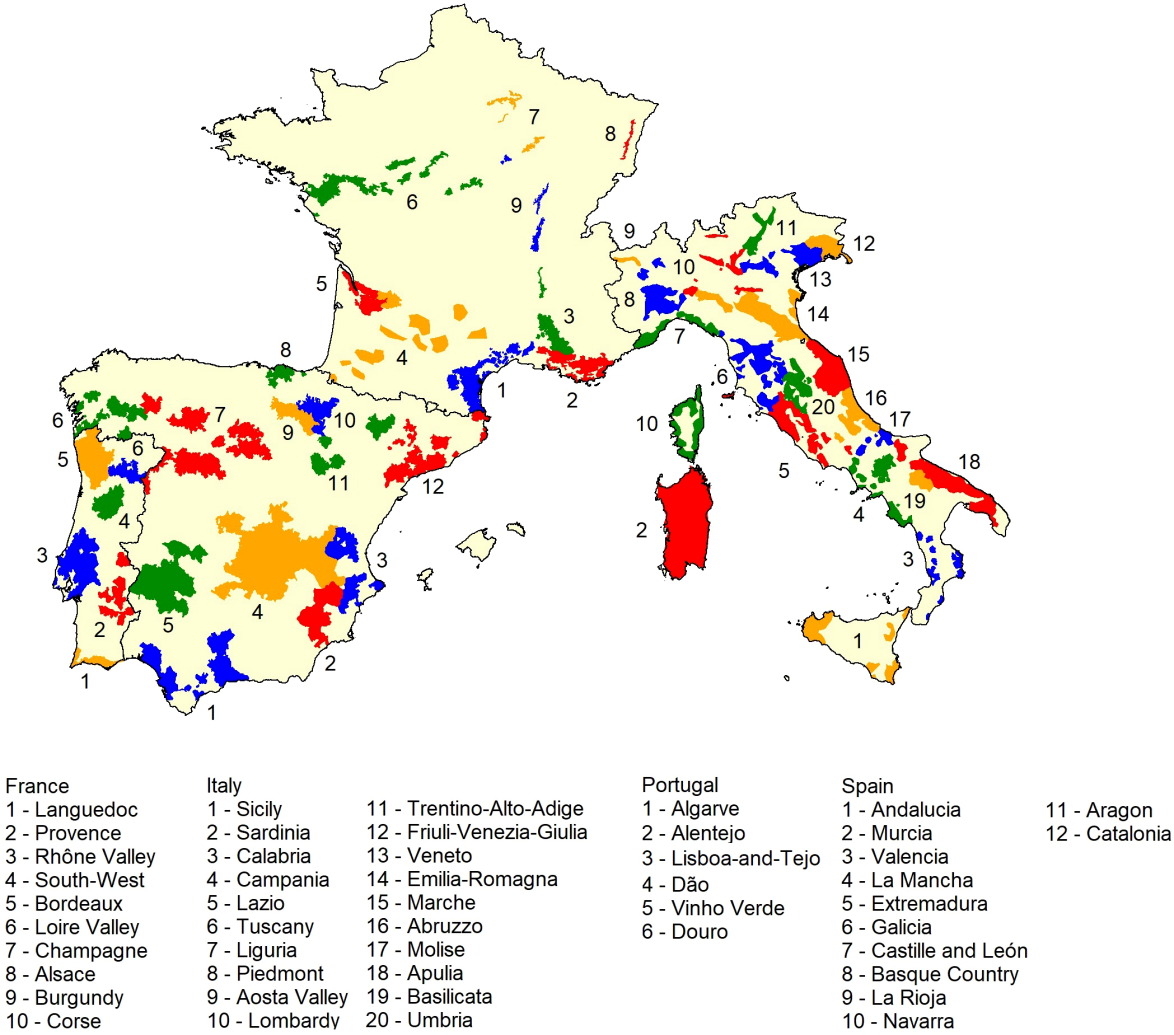
Study area and its main vinicultural zones (Fraga et al., 2020; Yang et al., 2022). Colors were used for distinctive purposes only.

The study area comprises the countries of France, Italy, Portugal, and Spain (**Figure 1**). Andorra and San Marino were included as well for the sake of data continuity, but will not be considered further in the study. The former countries have several renowned traditional vinicultural areas, which represent 25% of the world’s vineyards and grow hundreds of different grape varieties for wine-making (Santos et al., 2020; Eurostat, 2022; OIV, 2022). Some famous vinicultural regions include the Douro in Portugal, La Rioja in Spain, Bordeaux in France, and Tuscany in Italy. Furthermore, the corresponding wine production represents more than half of the world’s wine – red and white (OIV, 2022).

### 2.2 Grapevine varieties

**Figure 2:**
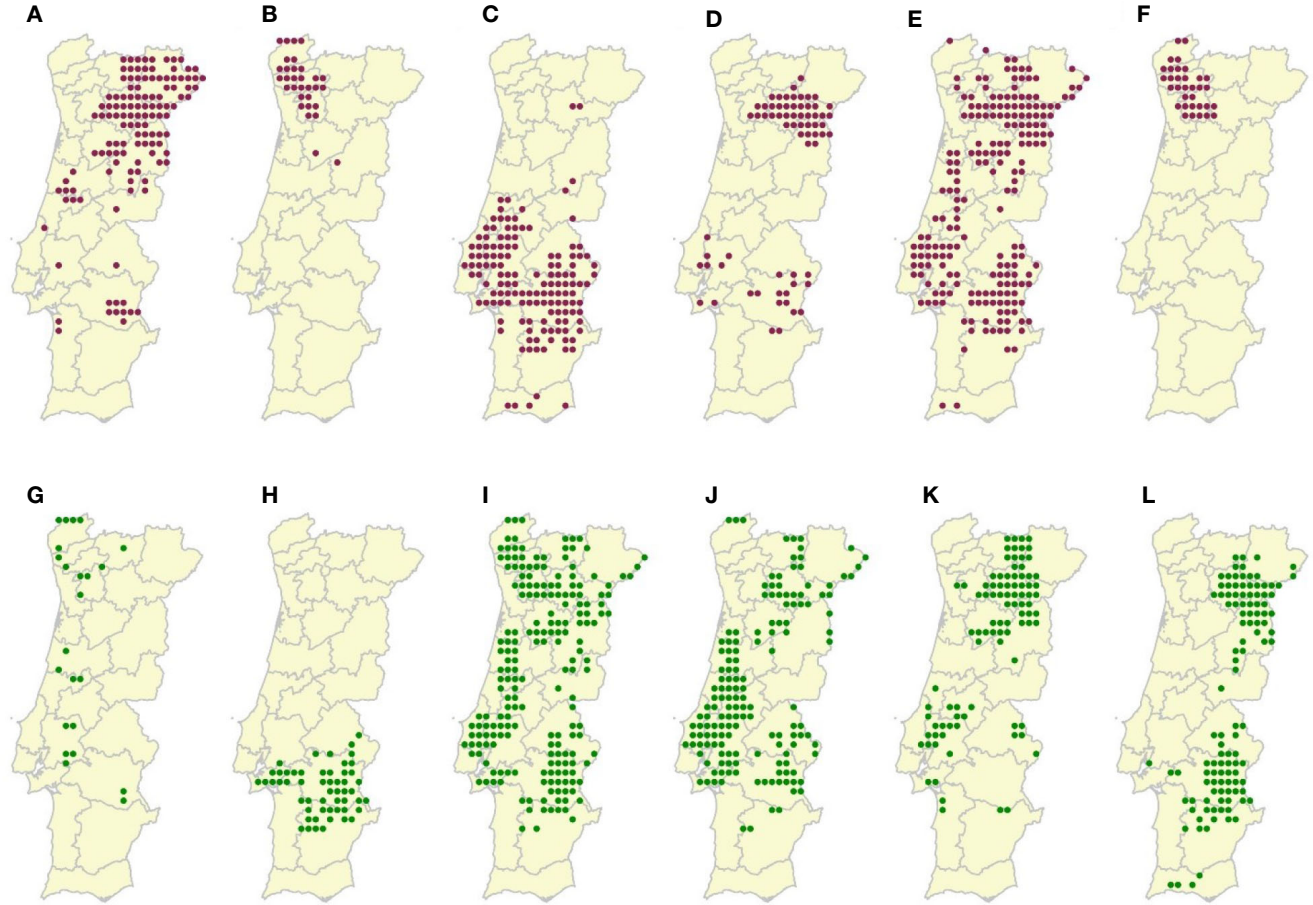
Sampled locations of the **(A)** Bastardo (Number of locations, N=123), **(B)** Borraçal (N=31), **(C)** Castelão (N=137), **(D)** Touriga-Franca (N=67), **(E)**Touriga-Nacional (N=202), **(F)** Vinhão (N=33), **(G)** Alvarinho (N=23), **(H)** Antão-Vaz (N=54), **(I)** Arinto (N=198), **(J)** Fernão-Pires (N=153), **(K)** Malvasia-Fina (N=92), and **(L)** Síria (N=113) grapevine varieties in mainland Portugal. Red dots represent red varieties and green dots represent white varieties.Polygons represent Portuguese NUTS 3 administrative units.

Twelve Portuguese (*Vitis vinifera*) grapevine varieties, both red and white, were chosen for this study (**Figure 2**). These varieties are endemic to Portugal and are adapted to different Portuguese regions and climates. Their most recent observed locations in Portugal were extracted from (Fraga et al., 2016) and sampled with the software QGIS 3.23.0 (Nightly), using a research rectangular grid, with a spatial resolution of 12.5 km, which matched the resolution of the climatic data selected for this study.

### 2.3 Climatic data

As stated previously in the introduction, the climatic conditions associated with the grapevine varieties were characterized by the “Cool Night index” (CN), the “Huglin Index” (HI), the “Temperature Range during Vine Ripening index” (TRR), and the “Growing Season Precipitation index” (GSP) bioclimatic indexes (**Table 1**). CN describes the temperature at night during September (ripening period), whilst HI describes heat accumulation by summing daily mean and maximum temperatures during the growing season (from April to September, in the northern hemisphere) and considers the length of the days for the highest latitudes. Both indices allow for a characterization of the climate of a particular region in terms of grape quality and wine properties, such as sugar content, acidity, color, or aroma (Tonietto and Carbonneau, 2004). As for the TRR and GSP indices, they describe the temperature ranges during the very sensitive grape berry maturation period and precipitation levels during the growing season, respectively. Additional indices were considered and calculated for this study, such as the Dryness Index (Tonietto and Carbonneau, 2004), but were excluded for two reasons, mainly because of high collinearity with chosen indices and negatively affecting the ENMs.

**Table 1:** - List of bioclimatic indices.

Index	Description	Units	Minimum Value	Maximum Value	Definition/Formula
CN	Cool Night Index	°C	-1.603	20.292	T_n September_
GSP	Growing Season Precipitation	mm	55.29	1166.40	$\sum_{01.04}^{31.09}\text{P}$
HI	Huglin Index	°C	0.864	3145.195	$\sum_{01.04}^{31.09}\frac{\left[\left(\text{T}-10\right)-\left({\text{T}}_{\text{x}}-10\right)\right]}{2}\text{d}$
TRR	Temperature Range during Ripening	°C	7.92	21.96	T_x September_ – T_n August_

P, Precipitation; T, Mean Temperature; T_x_, Maximum Temperature; T_n_, Minimum Temperature; d, length of day coefficient.

In the present study, two climatic datasets were used to compute the bioclimatic indices. Gridded daily mean, minimum and maximum air temperatures, and the daily total precipitation was retrieved from the E-OBS observational dataset (Cornes et al., 2018) and the EURO-CORDEX climate model simulation datasets (Jacob et al., 2014). The E-OBS gridded variables were bi-linearly interpolated into the coarser EURO-CORDEX grid to match its resolution of 12.5 km. The EURO-CORDEX data were retrieved from four regional climate models, namely, the ALADIN53, WRF331F, CCLM4-8-17, and REMO2009, which were forced by CNRM-CM5 (Voldoire et al., 2013), IPSL-CM5A-MR (Mignot and Bony, 2013), ICHEC-EC-EARTH (Döscher et al., 2022), and MPI-ESM (Giorgetta et al., 2013) the global climate models, respectively. These simulation data were driven by the two IPCC anthropogenic radiative forcing scenarios, RCP 4.5 and 8.5, to model climate change. The first scenario is more moderate in terms of greenhouse gas emissions, peaking in the mid-21^st^ century, while the second scenario corresponds to a more intensive fossil-fuel burning scenario, with much higher emissions and concentrations (IPCC, 2021).

Given that the climatic data provided by these models contain significant deviations from the observational datasets (both in mean and distribution), it is necessary to perform a preliminary treatment on these datasets. Therefore, the simulated datasets were bias-corrected by an empirical quantile mapping approach, as described in Cofiño et al. (2018), using the E-OBS dataset over the recent past period (1989–2005) as the baseline. This data treatment is standard regarding the application of climate model data (Martins et al., 2021).

After this procedure, the aforementioned bioclimatic indices were then computed for the recent past and future periods. The index values at each grid box or pixel correspond to the mean taken across the climatological normal, calculated for a recent past period (1989–2005) and two future periods (2021–2051 and 2051–2080). To evaluate collinearity between the indices, the Pearson correlation coefficients (PCC) and the variation inflation factors (VIF) were calculated. Collinearity can cause model uncertainty and should be assessed before proceeding to the modeling stage (de Marco and Nóbrega, 2018).

### 2.4 Ecological niche models

ENMs were built using the R modeling package BIOMOD2, version 3.5.1 (Thuiller et al., 2009). Firstly, a set of correlative models was obtained for each variety, using their current locations and the bioclimatic indices as inputs. Mandatory variety absence locations (locations where the varieties are not present) were generated randomly across the study area. Different numbers of absences were tested and 500 absences provided the best trade-off between the projected suitability and parsimony in the number of computations (**Figure S1**). On this matter, no consensus was found in the literature for the ideal number of absences to be used, so the authors decided upon a number that emphasized presence prevalence (number of presences per number of absences), following the recommendation from Santini et al. (2021).

The models were obtained using different modeling algorithms, namely the Generalized Additive Models [GAM, Hastie and Tibshirani (1990)], Generalized Boosting Model [GBM, Ridgeway (1999)], Classification Tree Analysis [CTA, Breiman et al. (2017)], Artificial Neural Networks [ANN, Ripley (1996)], and Random Forests [RF, Breiman (2001)] algorithms, with BIOMOD2 default settings. Other algorithms were tested as well, such as Generalized Linear Models [GLM, Nelder and Wedderburn (1972)] or Multivariate Adaptive Regression Splines [MARS, Friedman (1991)], but the choice fell on those that provided the most informative ensemble model projections. The models were trained and tested, using cross-validation on an 80/20 data partition scheme. This process was repeated 10 times, which led to the production of 50 models in total. Each one of them was evaluated for its performance using the area under the relative operating characteristic curve [AUC, Fielding and Bell (1997)], true skills statistic [TSS, Allouche et al. (2006)], and Cohen’s Kappa coefficient [KAPPA, Allouche et al. (2006)] metrics. The score interval for these metrics are [0; 1], [-1; 1] and [-1, 1], respectively, and scores above 0.8 are considered as good to excellent (Swets, 1988). Ensemble models were then built using all the ENMs with AUC scores above 0.8, taking the total consensus approach (Thuiller et al., 2009), with the weight of each one being defined by the weighted means of probabilities algorithm on its default setting. In turn, the ensemble models were evaluated by averaging the model scores for each metric. Ultimately, these models were used to spatially represent the bioclimatic suitability (expressed in terms of probability of occurrence) of the study area for each grape variety in the recent past and the future. In addition to the spatial representations of bioclimatic suitability, boxplots were built to evaluate the distribution of probabilities in the recent past and for each of the climatic scenarios, regarding both latitude and elevation, *via* pixel count (probabilities are contained in each pixel of the gridded data). These two factors are relevant for the study of the change of grapevine bioclimatic suitability related to climate change (Moriondo et al., 2013; Santos et al., 2020). A cutoff of 20% probability was chosen to make it possible a visual assertion of the potential changes between the recent past and the future. Lastly, the influences of each bioclimatic index on the ensemble models were assessed *via* permutation and by plotting the corresponding response curves. The response curves were obtained by assessing the response of each variety to each index, while the remaining ones were set to their mean value (Elith et al., 2005).

## 3 Results

### 3.1 Bioclimatic index maps

**Figure 3:**
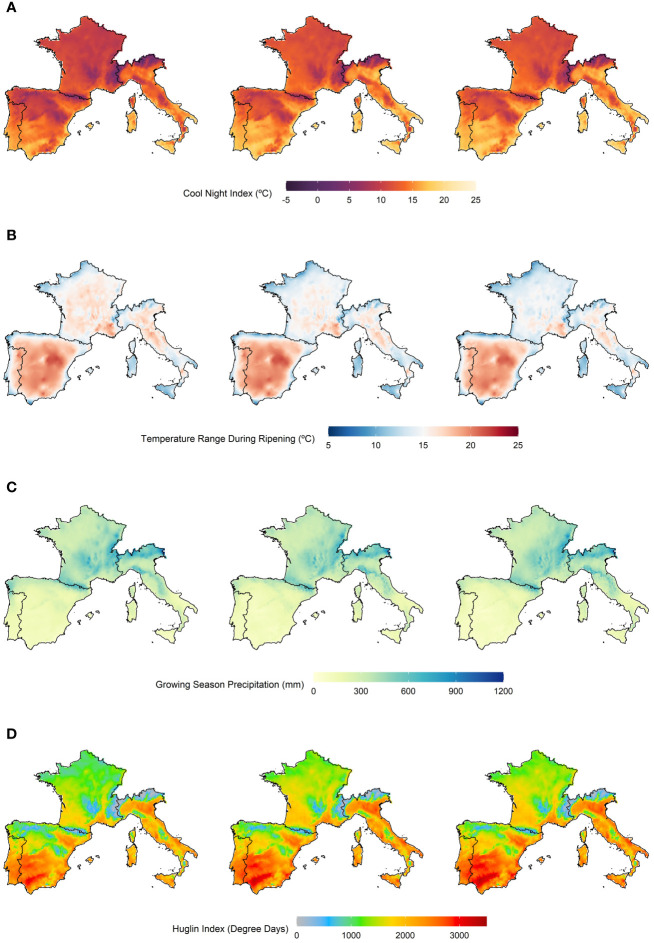
**(A)** Cool Night Index, **(B)** Temperature Range During Ripening Index, **(C)** Growing Season Precipitation Index, and **(D)** Huglin index. Each index was computed using the E-OBS dataset, for a recent-past period (1989–2005), and the EURO-CORDEX datasets, for the earlier future period (2021–2050), under the two climatic scenarios RCP 4.5 and 8.5. Left panels: Recent-past period. Middle panels: Future period, under the RCP 4.5 climatic scenario. Right panels: Future period, under the RCP 8.5 climatic scenario.

The computed bioclimatic indices, corresponding to the earlier future period 2021–2050, are presented in **Figure 3**. The values corresponding to the future climates, under RCP 4.5 and RCP 8.5, are medians taken across the indices calculated with each one of the four EURO-CORDEX datasets. The index values for the later future period (2051–2080) are available in the Supplementary Material (**Figure S3**).

Starting with the recent past period, the CN index showed the highest minimum temperatures in Portugal, Spain, and Italy (> 15 °C) and the lowest in France and Italy (< 15 °C), namely in the Alps. Furthermore, elevation has a clear effect on temperature, with major mountain ranges such as the Pyrenees or the Alps showing the lowest values. As for the TRR index, the temperature ranges are low in coastal areas (< 12 °C) and increasingly higher inland (> 12 °C). This is particularly the case for the Iberian Peninsula, where ranges vary from 8 to 22 °C. Some exceptions are observable in the southwest of France and the north and south of Italy, where ranges are higher than 10 °C in the coastal areas and lower than 12°C inland. Regarding the GSP index, the highest precipitation values are observed in France and the center-north of Italy (> 500 mm), while the lowest is observed in the south of Portugal and Spain (< 250 mm). The latter countries have higher precipitations in their northern regions (> 250 mm). Lastly, the HI indicates higher heat accumulation in the south of Spain (> 3000 degree days) and lower in areas of high elevation (< 500 degree days). In general, areas of low elevation have higher heat accumulation (> 2000 degree days), except for the center north of France, where insolation is lower. Regarding the earlier future period, in both RCP 4.5 and 8.5 climatic scenarios, values for the CN index increase some degrees across the whole study area. The TRR index values decreased in France and Italy, while the values for the GSP index increased in France, remain the same in Italy, and decrease in Portugal and Spain. Lastly, the values for the HI increase in the whole study area, although not dramatically.

### 3.2 Collinearity analysis

Literature on the topic of collinearity recommends selecting variables with scores below the thresholds of 0.7 for PCC (Dormann et al., 2012) and 10 for VIF (Neter et al., 1983). On one hand, the GSP and TRR indices have scored well below these thresholds and, therefore, have low collinearity with the other variables, which deems them fit for ENMs (**Table S1**). On the other hand, the CN and the HI indices do surpass the recommended PCC threshold regarding each other and surpass the VIF threshold as well. Despite this, it was decided not to exclude these indices from the modeling, as their information is fundamental to the objectives of this study (e.g. index ranking).

### 3.3 Index ranking and response curves

Index permutation to evaluate the influence of the indices in the modeling showed that the GSP index is the most influential of all indices in ensemble modeling (**Table S2**), ranking first for seven varieties. Mean scores vary between 0.177 and 0.983. The second most influential is the HI index, ranking first in the remaining five varieties. Mean scores were between 0.114 and 0.950. The remaining CN and TRR indices rank third and fourth, respectively.

As for the response curves, these showed that the probability of occurrence for most varieties is higher for the GSP index values lower than 250 mm (**Figure S2**). For the HI, the probability is consistently higher for values higher than 1500 degree days. Regarding the CN index, the probability of occurrence varies depending on the variety. For most, the probability is higher for values below 10 °C, sometimes peaking at this value, but for varieties Borraçal and Vinhão, probabilities are higher after it. Finally, the response curves for the TRR index showed that probabilities are generally higher for values below 16 °C.

### 3.4 Model evaluation

The initial set of correlative models obtained, overall, scores above 0.800 for AUC (**Table S3**). As for the TSS and KAPPA scores, they are generally lower, with values for TSS within the 0.700–0.800 interval and KAPPA within the 0.600–0.700 interval, except for Alvarinho, which has lower values for both metrics. The best-performing methods were GBM, GAM, and RF, with AUC scores ranging from 0.813 to 0.990. The methods CTA and ANN have AUC scores ranging from 0.742 to 0.985. The Antão-Vaz variety obtained the best model scores, while the Alvarinho variety obtained the worst.

Regarding the ensemble models, scores are significantly higher for all metrics when compared with the individual models (**Table 2**). AUC scores are higher than 0.982, TSS scores are higher than 0.851, and KAPPA scores are higher than 0.806. Antão-Vaz obtained the best scores, while Arinto obtained the worst.

**Table 2:** - Ensemble model metric scores for each grape variety.

White Variety	AUC	TSS	KAPPA	Red Variety	AUC	TSS	KAPPA
**Alvarinho**	0.994	0.954	0.810	**Bastardo**	0.983	0.900	0.810
**Antão-Vaz**	0.999	0.982	0.949	**Borraçal**	0.998	0.974	0.910
**Arinto**	0.982	0.866	0.847	**Castelão**	0.993	0.928	0.896
**Fernão-Pires**	0.985	0.892	0.862	**Touriga-Franca**	0.987	0.917	0.806
**Malvasia-Fina**	0.987	0.879	0.857	**Touriga-Nacional**	0.982	0.851	0.841
**Síria**	0.991	0.930	0.877	**Vinhão**	0.994	0.942	0.823

### 3.5 Ensemble model bioclimatic suitability

**Figure 4:**
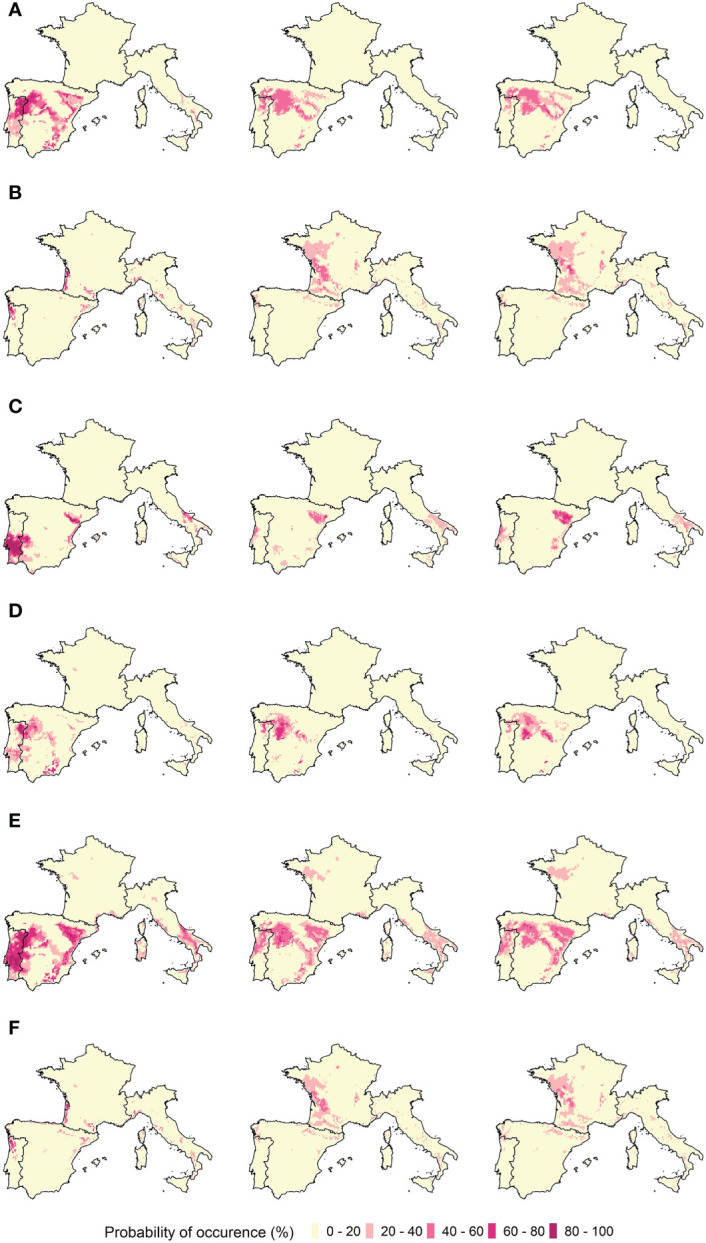
Ensemble model projected bioclimatic suitability for red varieties **(A)** Bastardo, **(B)** Borraçal, **(C)** Castelão, **(D)** Touriga-Franca, **(E)** Touriga-Nacional, and **(F)** Vinhão. Left panels: Recent past distribution of bioclimatic suitability (1989–2005). Middle panels: Future bioclimatic suitability projections (2021–2050), under the RCP 4.5 climatic scenario. Right panels: Future bioclimatic suitability projections (2021–2050), under the RCP 8.5 climatic scenario.

**Figure 5:**
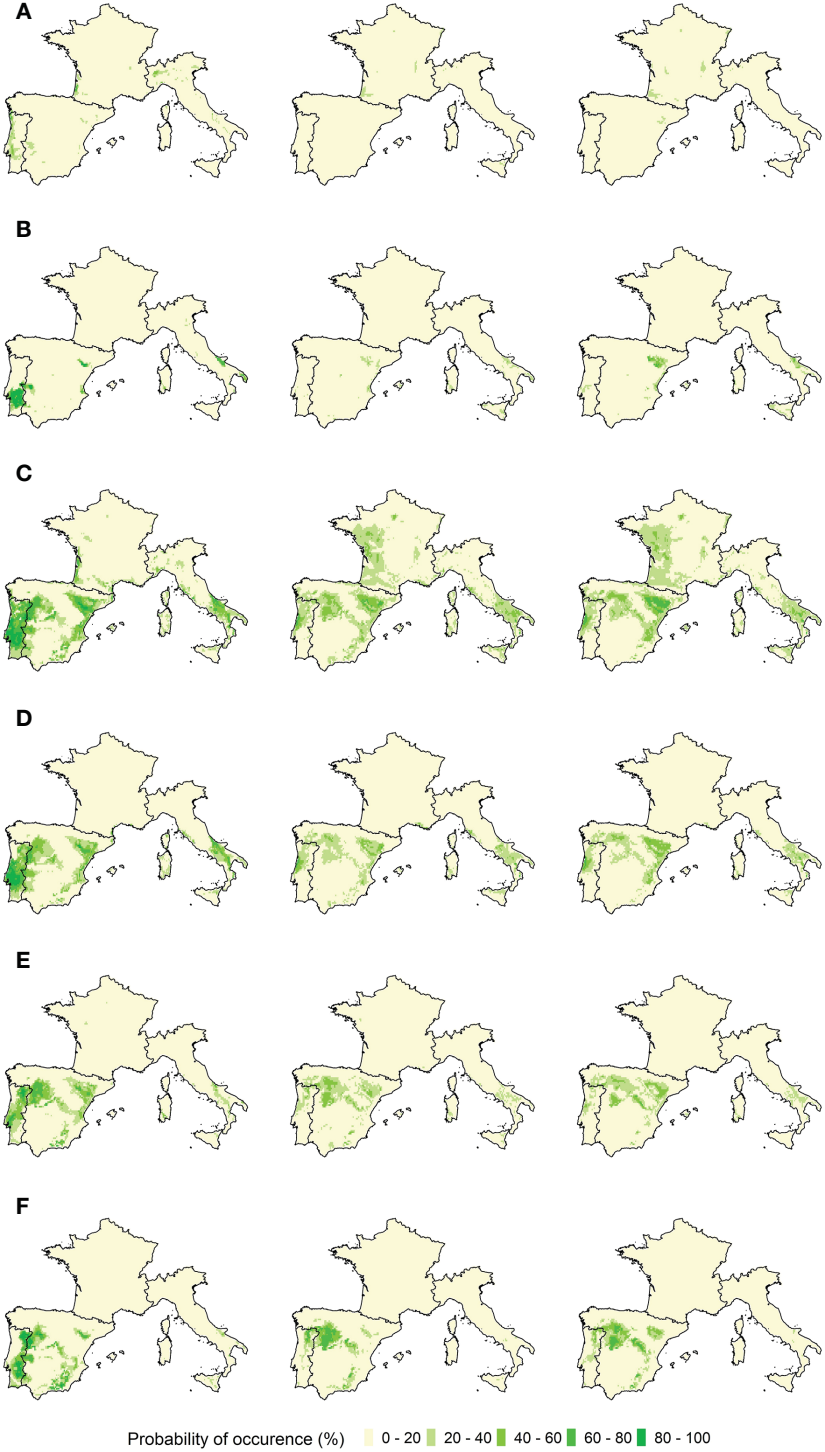
Ensemble model projected bioclimatic suitability for white varieties **(A)** Alvarinho, **(B)** Antão-Vaz, **(C)** Arinto, **(D)** Fernão-Pires, **(E)** Malvasia-Fina, and **(F)** Síria. Left panels: Recent past distribution of bioclimatic suitability (1989–2005). Middle panels: Future bioclimatic suitability projections (2021–2050), under the RCP 4.5 climatic scenario. Right panels: Future bioclimatic suitability projections (2021–2050), under the RCP 8.5 climatic scenario.

The ensemble model results for each of the grapevine varieties are presented in **Figures 4**, **5**. Bioclimatic suitability distributions were estimated for the recent past (1989–2005) and projected to the earlier future period (2021–2050), for both RCP 4.5 and 8.5 climatic scenarios. The projected bioclimatic suitability for the later future period (2051–2080) can be found in the Supplementary Material (**Figures S4–S5**).

The red varieties Bastardo, Castelão, Touriga-Franca, and Touriga-Nacional have bioclimatic suitability in the Alentejo, Dão, Lisboa-and-Tejo, and Douro regions in Portugal, the Andalucia, Aragon, Castille and Léon, Catalonia, Extremadura, La Mancha, La Rioja, Murcia, Navarra, and Valencia regions in Spain, and the Apulia and Basilicata regions in Italy. As for the varieties Borraçal and Vinhão, they have bioclimatic suitability mainly in the Minho region in Portugal and the Bordeaux region in France, and also partly in the Catalonia region in Spain and the Liguria region in Italy. In the case of white varieties, Antão-Vaz, Arinto, Fernão-Pires, Malvasia-Fina, and Síria varieties have bioclimatic suitability mainly in the Alentejo, Dão, Lisboa-and-Tejo, and Douro regions in Portugal, the Andalucia, Aragon, Extremadura, Castille and Léon, Navarra, Catalonia, La Mancha, Murcia, and Valencia regions in Spain, and the Apulia, Basilicata and Campania regions in Italy. Alvarinho is the only exception, with its bioclimatic suitable areas located mainly in the Portuguese coastal areas, the Bordeaux region in France, and the Aosta Valley in Italy. The Bordeaux bioclimatic suitability for Alvarinho is shared with that of Arinto.

Regarding future projections, the bioclimatic suitability of the red varieties Borraçal and Vinhão moved completely to France and the north of Spain and remains residually in Italy and Portugal. New suitable areas now include the French regions of Alsace, Burgundy, Champagne, Loire Valley, and South-West, and the Spanish regions of Basque Country, Galicia, and Navarra. Bioclimatic suitability for varieties Bastardo, Castelão, Touriga-Franca, and Touriga-Nacional shifted towards the north of Portugal and Spain, and are located in the Aragon, Asturias, Basque Country, Catalonia, Dão, Douro, Galicia, Lisboa-and-Tejo, and Navarra regions. These varieties are no longer suitable in the Alentejo and Extremadura regions and remain so residually in Andalucia. In Italy, there is a generalized loss of suitability. In the case of white varieties, developments are similar. Arinto’s bioclimatic suitability is more diversified in France and is now presented as suitable in the regions of Alsace, Burgundy, Champagne, Loire Valley, and Provence. It is no longer suitable in the Bordeaux region, however. In Portugal and Spain, bioclimatic suitability for Arinto, Fernão-Pires, Malvasia-Fina, and Síria shifts towards the northern regions of Aragon, Castille and León, Basque Country, Dão, Douro, La Rioja, Navarra, and Galicia, and is no longer present in the Alentejo, Andalucia, Extremadura, and Lisboa-and-Tejo regions. Antão-Vaz’s bioclimatic suitability remains in the Aragon, La Rioja, and Navarra regions, but is no longer present in Portugal. In Italy, Antão-Vaz, Arinto, Fernão-Pires, and Malvasia-Fina lose bioclimatic suitability, whilst Síria no longer has it.

As for the later future period, the previous developments continue in the same direction: further shift of the bioclimatic suitability towards the northern regions of the study area, more so under the RCP 8.5 scenario. This is especially the case for red varieties Borraçal and Vinhão, and white variety Arinto, with respective bioclimatic suitabilities shifting to the French regions of Alsace and Champagne. Under the RCP 4.5 scenario, there is not a pronounced change in comparison with the earlier future period.

**Figure 6:**
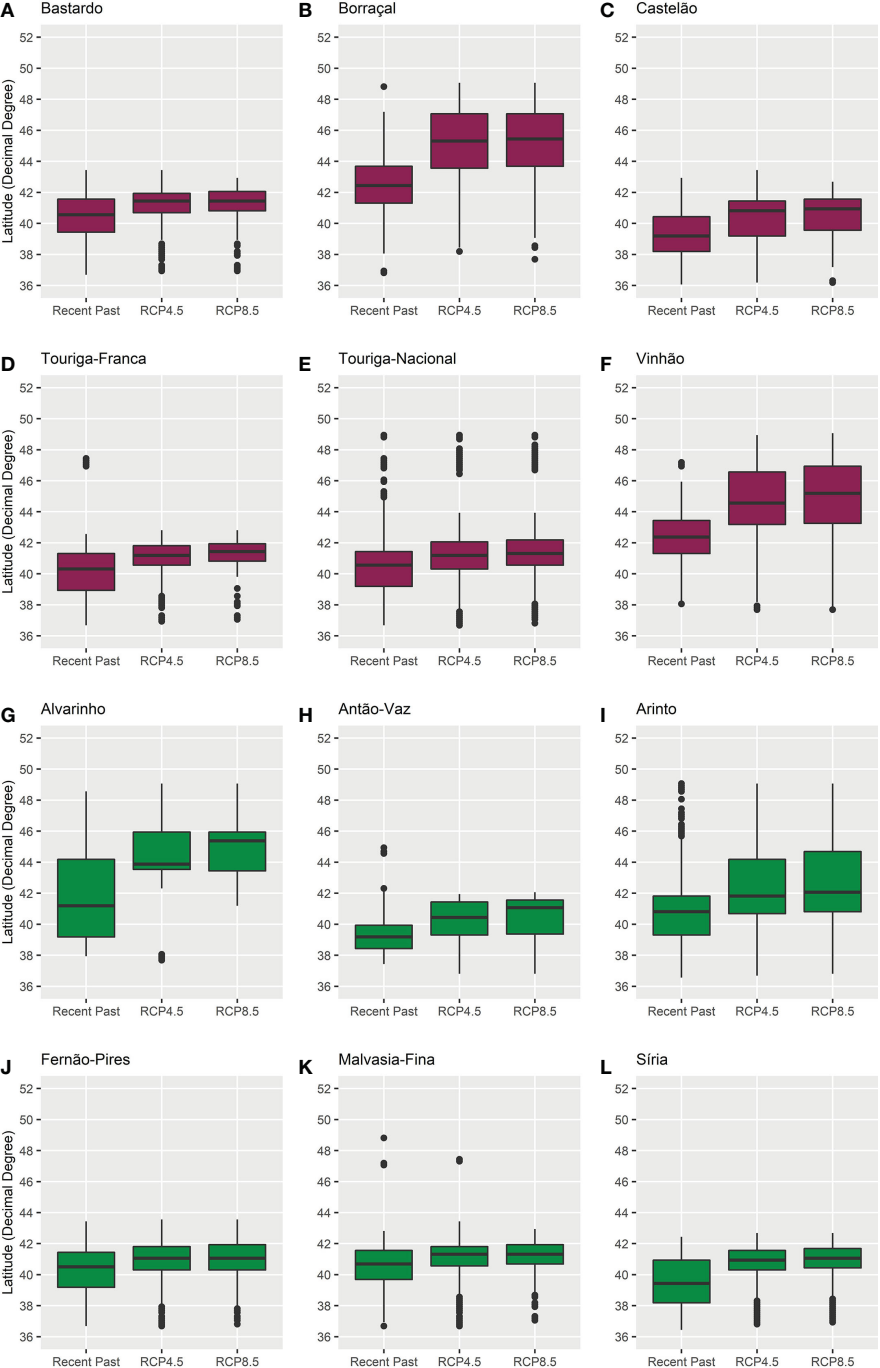
Boxplots of the distribution of bioclimatic suitability, above 20% probability of occurrence, in regards to latitude, for varieties **(A)** Bastardo, **(B)** Borraçal, **(C)** Castelão, **(D)** Touriga-Franca, **(E)** Touriga-Nacional, **(F)** Vinhão, **(G)** Alvarinho, **(H)** Antão-Vaz, **(I)** Arinto, **(J)** Fernão-Pires, **(K)** Malvasia-Fina, and **(L)** Síria, in the recent past (1989–2005) and future (2021–2050) climates. Red boxplots identify red varieties and green boxplots identify white varieties.

**Figure 7:**
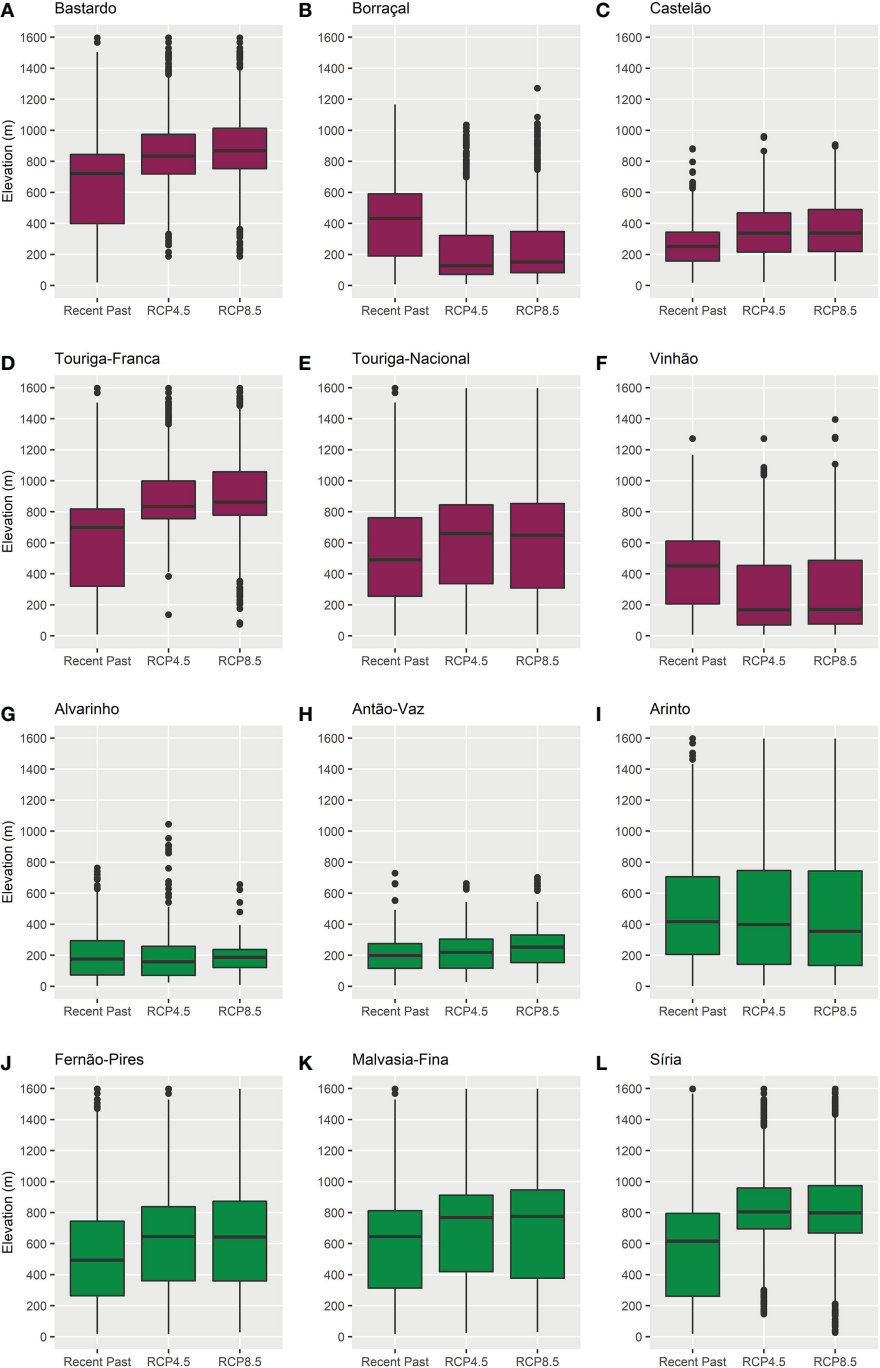
Boxplots of the distribution of bioclimatic suitability, above 20% probability of occurrence, in regards to elevation, for varieties **(A)** Bastardo, **(B)** Borraçal, **(C)** Castelão, **(D)** Touriga-Franca, **(E)** Touriga-Nacional, **(F)** Vinhão, **(G)** Alvarinho, **(H)** Antão-Vaz, **(I)** Arinto, **(J)** Fernão-Pires, **(K)** Malvasia-Fina, and **(L)** Síria, in the recent past (1989–2005) and future (2021–2050) climates. Red boxplots identify red varieties and green boxplots identify white varieties.

To improve the analysis of the ensemble model results, boxplots were built to evaluate the distribution of the bioclimatic suitability with a probability of occurrence above 20% regarding latitude and elevation (**Figures 6**, **7**). These showed that the median bioclimatic suitability increased from latitudes around 42 to 44 decimal degrees, establishing a positive correlation between latitude and the different climatic scenarios. The same is observed for elevation: median values increased in future climates, except for Borraçal and Vinhão, which decreased. The boxplots in the later future period (2051–2080) can be found in the Supplementary Material (**Figures S6–S7**).

## 4 Discussion

### 4.1 Impact of climate change on viniculture

In this study, four bioclimatic indices (CN, GSP, HI, and TRR) were determined for the recent past and IPCC’s climate change RCP 4.5 and 8.5 scenarios and similar conclusions can be drawn from them to those included in studies mentioned in the introduction (Luedeling et al., 2011; Fraga and Santos, 2021). However, a step further was taken with their application in building ENMs. Using them as predictor variables, correlative ensemble models were produced from individual ENMs - stemming from different modeling methods - to estimate the bioclimatic suitability of France, Italy, Portugal, and Spain for twelve Portuguese grape varieties, in the recent past climate (1989–2005) and the future (2021–2050 and 2051–2080). The importance of the indices to the modeling was evaluated and those related to the growing season precipitation and heat accumulation were the most influential (**Table S2**). This corroborates the importance of these two variables in the choice of appropriate locations for vineyards, as they are both highly influential in grapevine phenology (Jones, 2007; van Leeuwen and Darriet, 2016; Santos et al., 2020). According to the response curves, the probability of occurrence is higher for lower precipitation and higher heat accumulation (**Figure S2**). This means that the studied varieties should not need high amounts of water during the growing season to develop high-quality grapes. Conversely, considerable heat accumulation seems crucial for the proper phenological development of plants. Also, the obtained models showed high scores for different model performance evaluation metrics (**Table 2**), thus warranting that the chosen indices are highly informative predictor variables.

Model results must provide plausible distributions of the bioclimatic suitability of the species being studied (Guisan and Zimmermann, 2000). Considering the results for the recent past period, bioclimatic suitability is located in the areas where the varieties are currently present and in their close vicinity (**Figures 4**, **5**). This suggests current varietal suitability in Portugal could potentially be expanded to include more vineyards. Furthermore, other regions with similar climates were also identified. Starting with the red varieties Borraçal and Vinhão, suitable regions include the Minho region in Portugal and the Bordeaux region in France. These regions are humid coastal areas, with low-temperature ranges, as well as moderate heat accumulation and precipitation. Furthermore, for Bastardo and Touriga-Franca varieties suitable regions include the Douro region and several regions in Spain that have high-temperature ranges, high heat accumulation, and low precipitation, much like the Douro region. The same patterns are observed for the white varieties, depending on their locations. Taking Arinto as an example, this is a variety that is well adapted to various climates in Portugal. As a result, its bioclimatic suitability is estimated for several regions within the study area. When looking at the future projections, bioclimatic suitability has a northward and elevation shift, and for some varieties, new projected areas are observed in the north of France. This is a plausible result because of the projected rise in temperature and precipitation reduction in the southern part of the study area (IPCC, 2021), it is expected that the current climatic conditions associated with the studied varieties will be met further north and at higher elevations (Moriondo et al., 2013; Santos et al., 2020). As a final observation, projected bioclimatic suitability for Borraçal and Vinhão varieties do not shift in elevation as much as others, like Touriga-Nacional or Arinto, as they do not always find similar conditions in these areas to those they have now in the Minho region. This could be because higher elevations do not always translate to low-temperature ranges or increased precipitation.

### 4.2 Adaptation to climate change

The results presented herein can contribute to better planning of the vinicultural practices in Europe in the long term. On one hand, massive bioclimatic suitability shifts from the south to the north of Europe will mean major negative economic impacts on the vinicultural socioeconomic sector in Portugal, Spain, and Italy if no adaptation measures are taken. To name a few, associated with the selection of grapevine varieties (e.g., more heat and drought-tolerant), measures such as delaying the ripening period by the use of specific training systems, as well as minimal pruning systems, should be adopted. Furthermore, a reduction of radiation exposure, e.g. by installing shading systems, changing row orientation, and wind exposure, would improve temperature regulation. Moreover, higher water use efficiency will be very important as water demand will tend to increase in the future, due to economic and populational factors, despite its generally lower availability. On the other hand, countries in Central and Eastern Europe could start asserting which areas in their territory are viable for grapevine cultivation on a wider scale since bioclimatic suitability will likely increase in their territory, whilst taking care of implementing grape production policies that ensure the sustainable exploration of the land, low CO_2_ emissions, and biodiversity in the affected ecosystems.

### 4.3 Limitations and model uncertainties

Despite the validity of the results supported by the high evaluation metrics of the model performance, there are methodological limitations that should be contemplated. First, the reduced number of available locations for some varieties was influential in the respective model results. For instance, the AUC ensemble model evaluation of the Alvarinho variety suggests a case of model overfitting. Consequently, the model projected suitability was residual for the recent past climate and non-existent for the future climate scenarios, confirming that model performance is strongly influenced by the number of presences (Breiner et al., 2015). In the future, the models for some varieties (e.g. Alvarinho, Antão-Vaz) could be built using a more complete dataset to improve the model quality. Second, only one set of randomly generated absences was used for each variety, and using different sets of absences would improve the overall quality of model predictions. Third, it is generally recommended to use a minimum of five predictor variables (Guisan and Zimmermann, 2000). For the present study, additional indices were calculated but they had high levels of collinearity with the indices, which resulted in poorer modeling performance. Lastly, the resolution of the E-OBS and EURO-CORDEX did not allow for finer discrimination of the projected bioclimatic suitability. Future studies should focus on analyzing bioclimatic suitability at finer resolutions.

## 5 Conclusions

ENMs were produced for twelve Portuguese grape varieties to estimate the respective bioclimatic suitability in four wine-producing European countries (France, Italy, Portugal, and Spain) in the recent past and future IPCC RCP 4.5 and 8.5 climates, using BIOMOD2. To characterize the different climates, four bioclimatic indices were calculated. These indicated, for future scenarios, an increase in heat accumulation, throughout the study area, and a reduction of precipitation in its southern regions. Furthermore, the indices proved to be suitable predictor variables as the produced ENMs had, overall, good to excellent performance and their respective ensembles are very informative. Looking at the results, for the recent past climate, bioclimatic suitability is well distributed around the current locations of the modeled grapevine varieties in Portugal and is also estimated for other regions in the study area with similar climatic conditions. Compared with the future climates, the projected bioclimatic suitability of most varieties shifted towards the north of Spain and France, while some remained residually in Italy and Portugal. Projections also moved, in most cases, to areas with higher elevations. These results suggest winegrowers in southern Europe will most probably have to mitigate the effects of the projected changes to the most relevant atmospheric factors, namely heat accumulation, and precipitation, to ensure some degree of sustainability to their activity. In this regard, the use of ensembles of ENMs to study the bioclimatic suitability of grapevine varieties in Europe proved to be a valid way to understand the potential impact of climate change on the vinicultural socioeconomic sector in Europe in the decades to come.

## Data availability statement

The original contributions presented in the study are included in the article/**Supplementary Material**. Further inquiries can be directed to the corresponding author.

## Author contributions

Conceptualisation: all authors. Methodology and investigation: all authors. Software, validation, formal analysis, and data curation: FA, JC, HF. Writing—original draft preparation: FA. Writing—review and editing: all authors. Visualization and supervision: JS and AM. Project administration and funding acquisition: JS and AM. All authors have read and agreed to the published version of the manuscript. FA led the study and performed all of the tasks mentioned in the manuscript, such as data processing, ecological niche modeling, results analysis and interpretation. JC provided technical and scientific support with the ecological niche modeling. JS, AM, and HF provided scientific support on the topics of climate change and viniculture, and the necessary data to conduct the study. All authors contributed to the article and approved the submitted version.
