# Corrigendum: Relocation of bioclimatic suitability of Portuguese grapevine varieties under climate change scenarios

**DOI:** 10.3389/fpls.2023.1232948

**Published:** 2023-07-24

**Authors:** Filipe Adão, João C. Campos, João A. Santos, Aureliano C. Malheiro, Hélder Fraga

**Affiliations:** ^1^ Centre for the Research and Technology of Agro-Environmental and Biological Sciences (CITAB), University of Trás-os-Montes e Alto Douro (UTAD), Vila Real, Portugal; ^2^ Centre for Research in Geospace Science (CICGE), University of Porto, Vila Nova de Gaia, Portugal

**Keywords:** viniculture, future climate, ecological niche models, ensemble modeling, grape varieties, Europe, BIOMOD 2

**Error in Figure/Table**

In the published article, there was an error in the Figure 2 caption as published. The letters in the caption corresponding to the white and red grape varieties were switched.

Caption as published:

Figure 2 Sampled locations of the **(A)** Alvarinho (number of locations, N=23), **(B)** Antão-Vaz (N=54), **(C)** Arinto (N=198), **(D)** Fernão-Pires (N=153), **(E)** Malvasia-Fina (N=92), **(F)** Síria (N=113), **(G)** Bastardo (N=123), **(H)** Borraçal (N=31), **(I)** Castelão (N=137), **(J)** Touriga-Franca (N=67), **(K)** Touriga-Nacional (N=202), and **(L)** Vinhão (N=33) grapevine varieties in mainland Portugal. Green dots represent white varieties and red dots represent red varieties. Polygons represent Portuguese NUTS 3 administrative units.

**Figure 2** and its corrected caption appear below.

**Figure 2:**
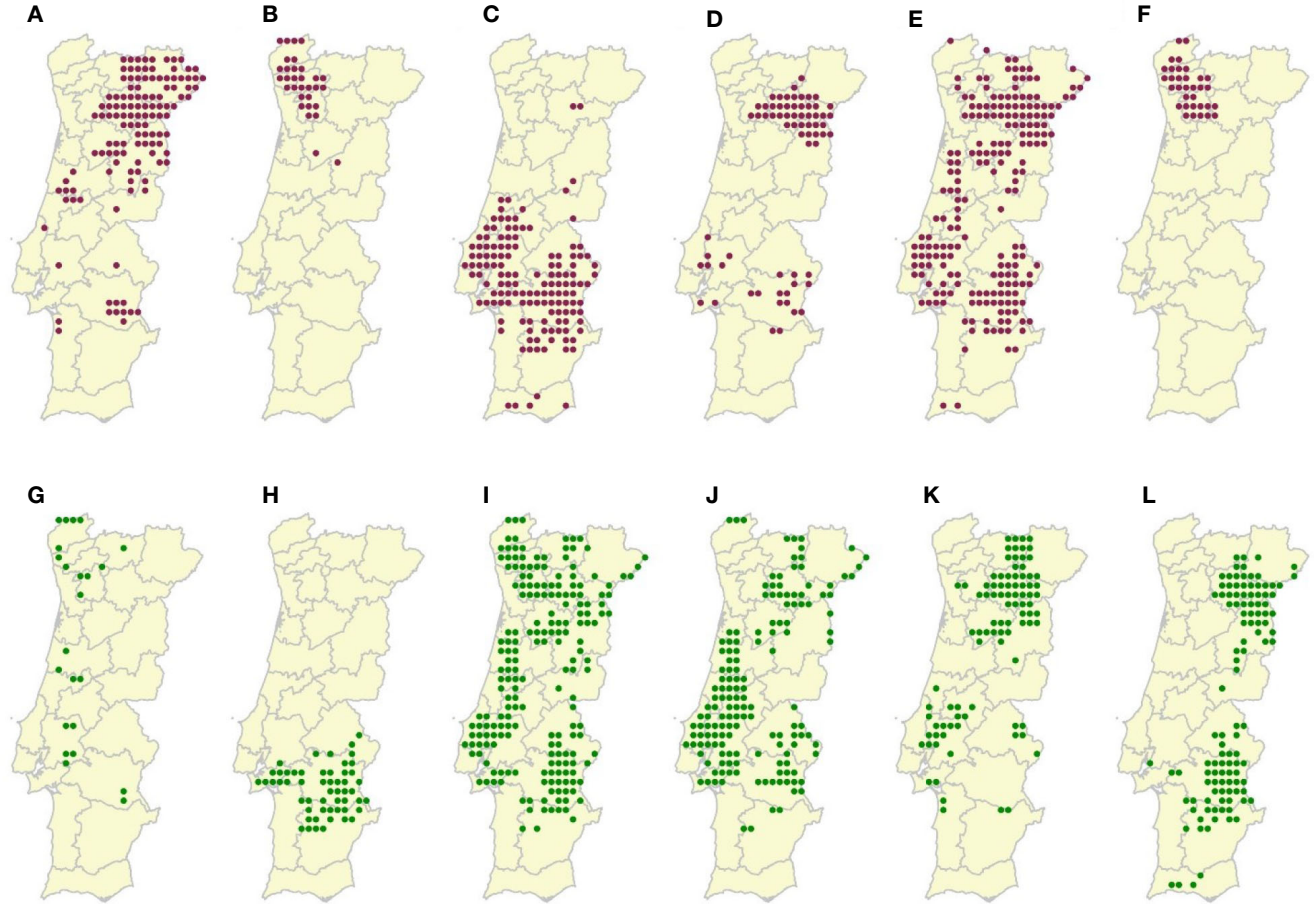
Sampled locations of the **(A)** Bastardo (Number of locations, N=123), **(B)** Borraçal (N=31), **(C)** Castelão (N=137), **(D)** Touriga-Franca (N=67), **(E)** Touriga-Nacional (N=202), **(F)** Vinhão (N=33), **(G)** Alvarinho (N=23), **(H)** Antão-Vaz (N=54), **(I)** Arinto (N=198), **(J)** Fernão-Pires (N=153), **(K)** Malvasia-Fina (N=92), and **(L)** Síria (N=113) grapevine varieties in mainland Portugal. Red dots represent red varieties and green dots represent white varieties. Polygons represent Portuguese NUTS 3 administrative units.

Corrected Caption:

The authors apologize for this error and state that this does not change the scientific conclusions of the article in any way. The original article has been updated.

