# Supplementary material for: Relocation of bioclimatic suitability of Portuguese grapevine varieties under climate change scenarios
Source: Front Plant Sci. 2023 Feb 8;14:974020. doi: 10.3389/fpls.2023.974020 (PMC9945296; doi:10.3389/fpls.2023.974020)
Supplement: Supplementary file 1 [file DataSheet_1.docx]

**Supplementary Material**

**
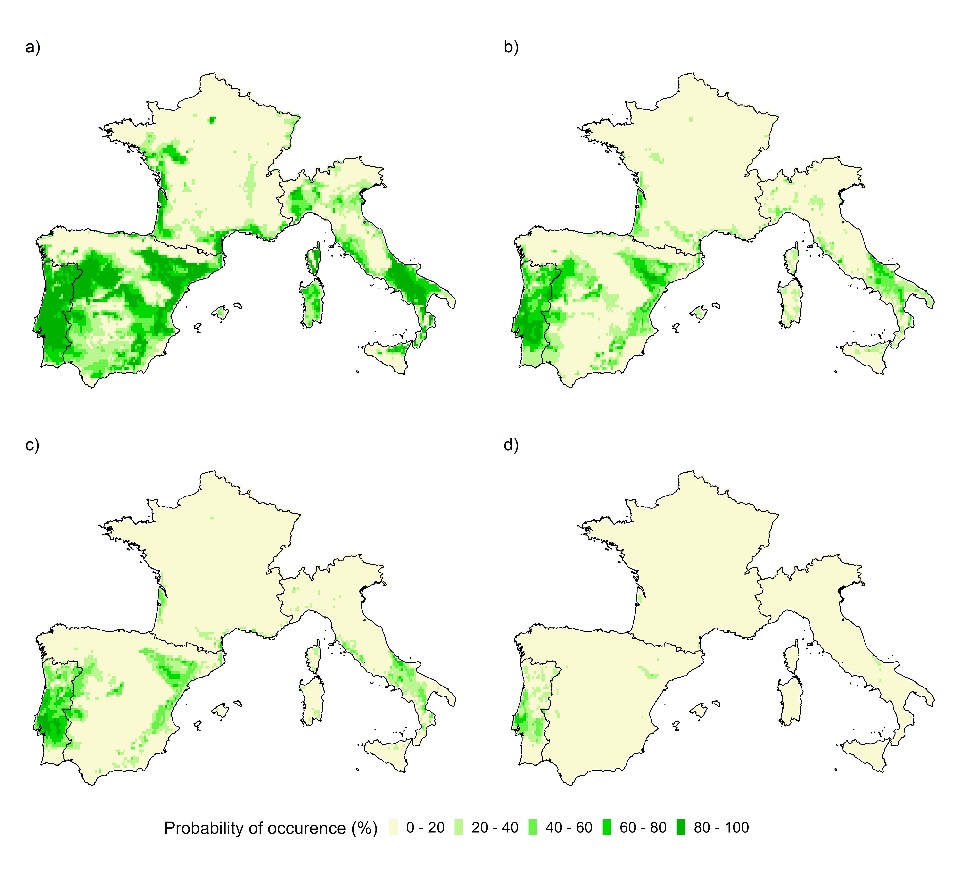
**

**Figure S1** – Ensemble model estimated bioclimatic suitability for white variety Arinto, respective to the recent past period (1989–2005), obtained using a) 100, b) 500, c) 1000, and d) 5000 randomly generated absences.





**Figure S2** – Index response curves for grapevine varieties a) Bastardo, b) Borraçal, c) Castelão, d) Touriga-Franca, e) Touriga-Nacional, f) Vinhão, g) Alvarinho, h) Antão-Vaz, i) Arinto, j) Fernão-Pires, k) Malvasia-Fina, and l) Síria.


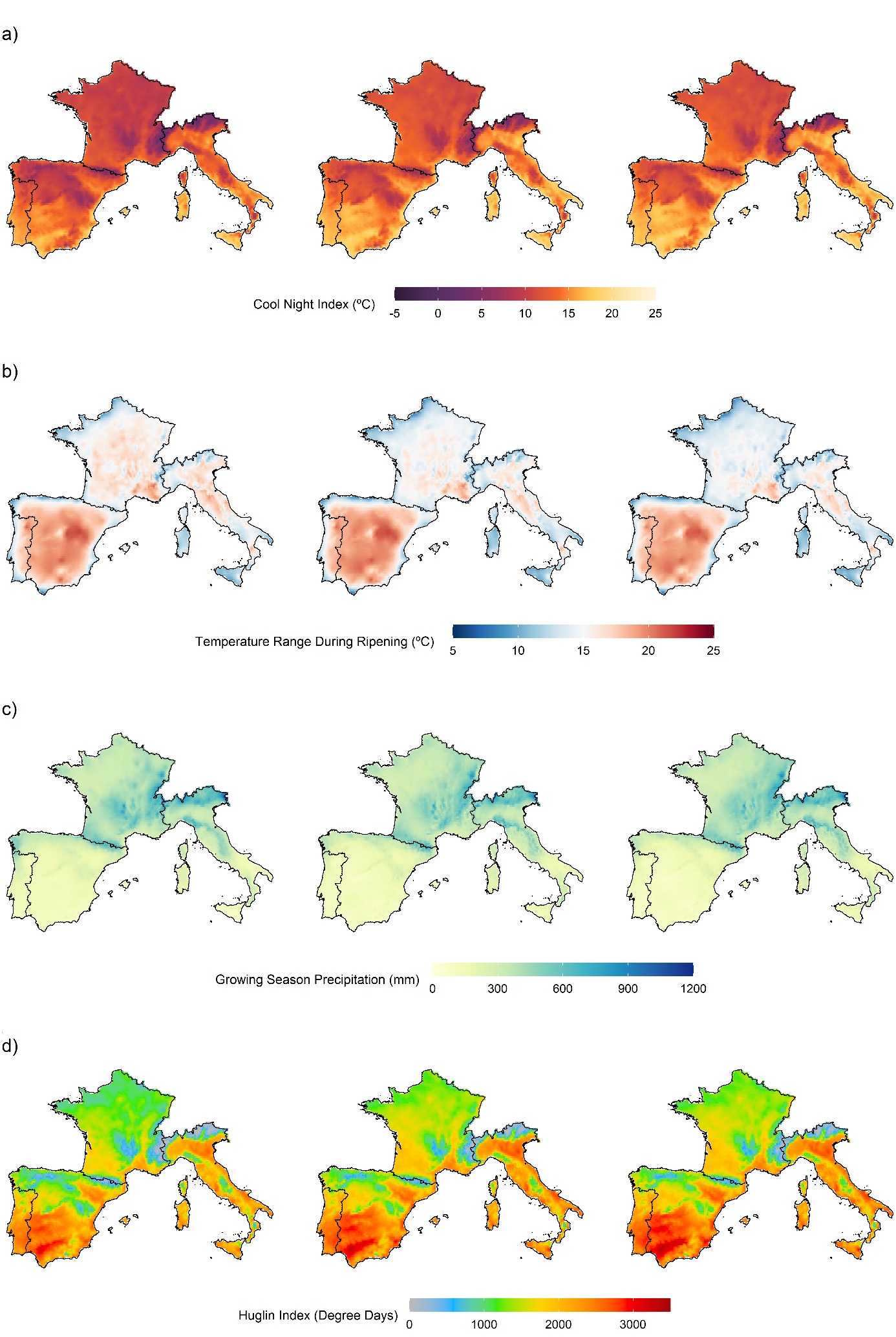


**Figure S3** – a) Cool Night Index, b) Temperature Range During Ripening Index, c) Growing Season Precipitation Index, and d) Huglin index. Each index was computed using the E-OBS dataset, for a recent-past period (1989–2005), and the EURO-CORDEX datasets, for the later future period (2051–2080), under the two climatic scenarios RCP 4.5 and 8.5. Left panels: Recent-past period. Middle panels: Future period, under the RCP 4.5 climatic scenario. Right panels: Future period, under the RCP 8.5 climatic scenario.


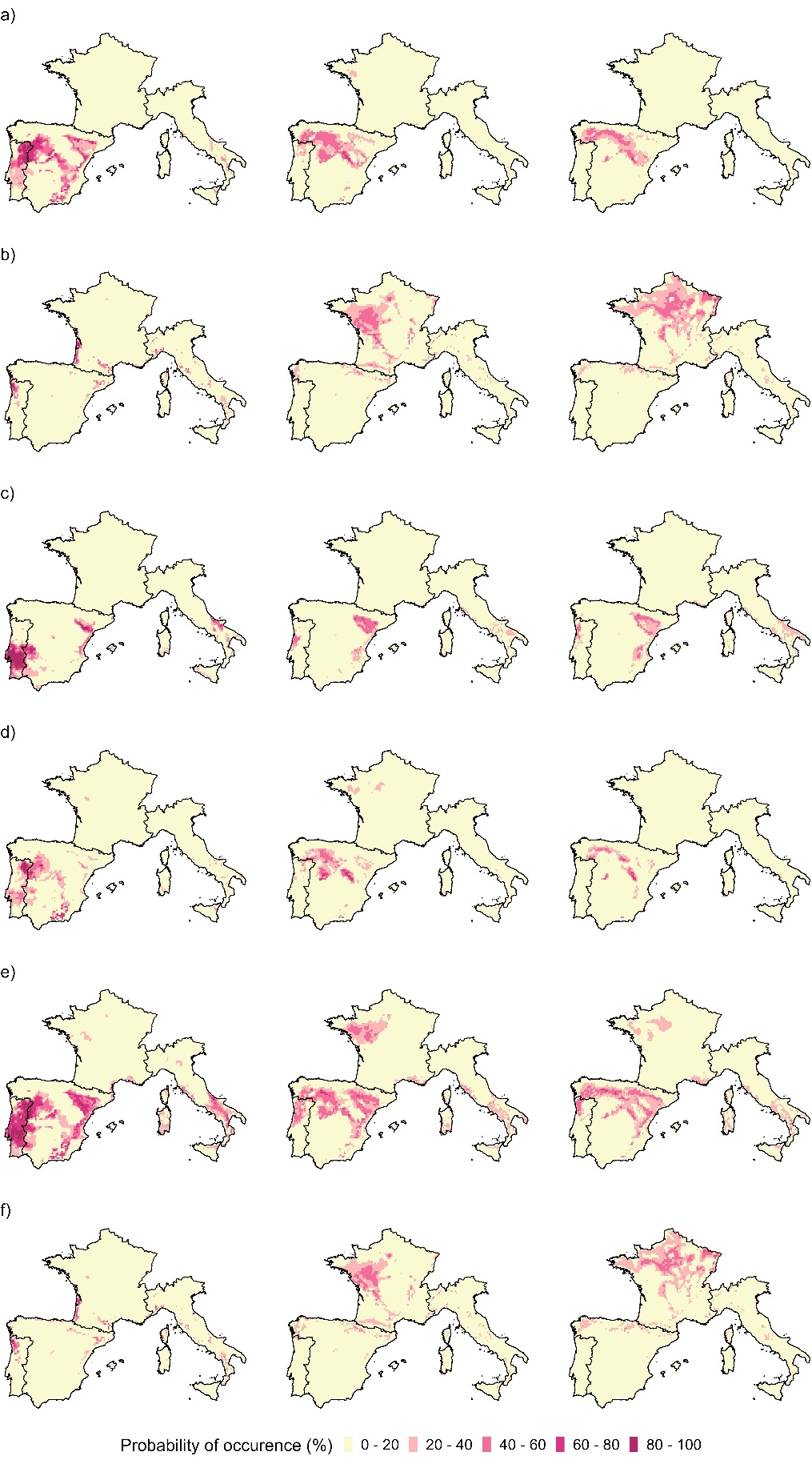


**Figure S4** – Ensemble model projected bioclimatic suitability for red varieties a) Bastardo, b) Borraçal, c) Castelão, d) Touriga-Franca, e) Touriga-Nacional, and f) Vinhão. Left panels: Recent past distribution of bioclimatic suitability (1989–2005). Middle panels: Future bioclimatic suitability projections (2051–2080), under the RCP 4.5 climatic scenario. Right panels: Future bioclimatic suitability projections (2051-2080), under the RCP 8.5 climatic scenario.


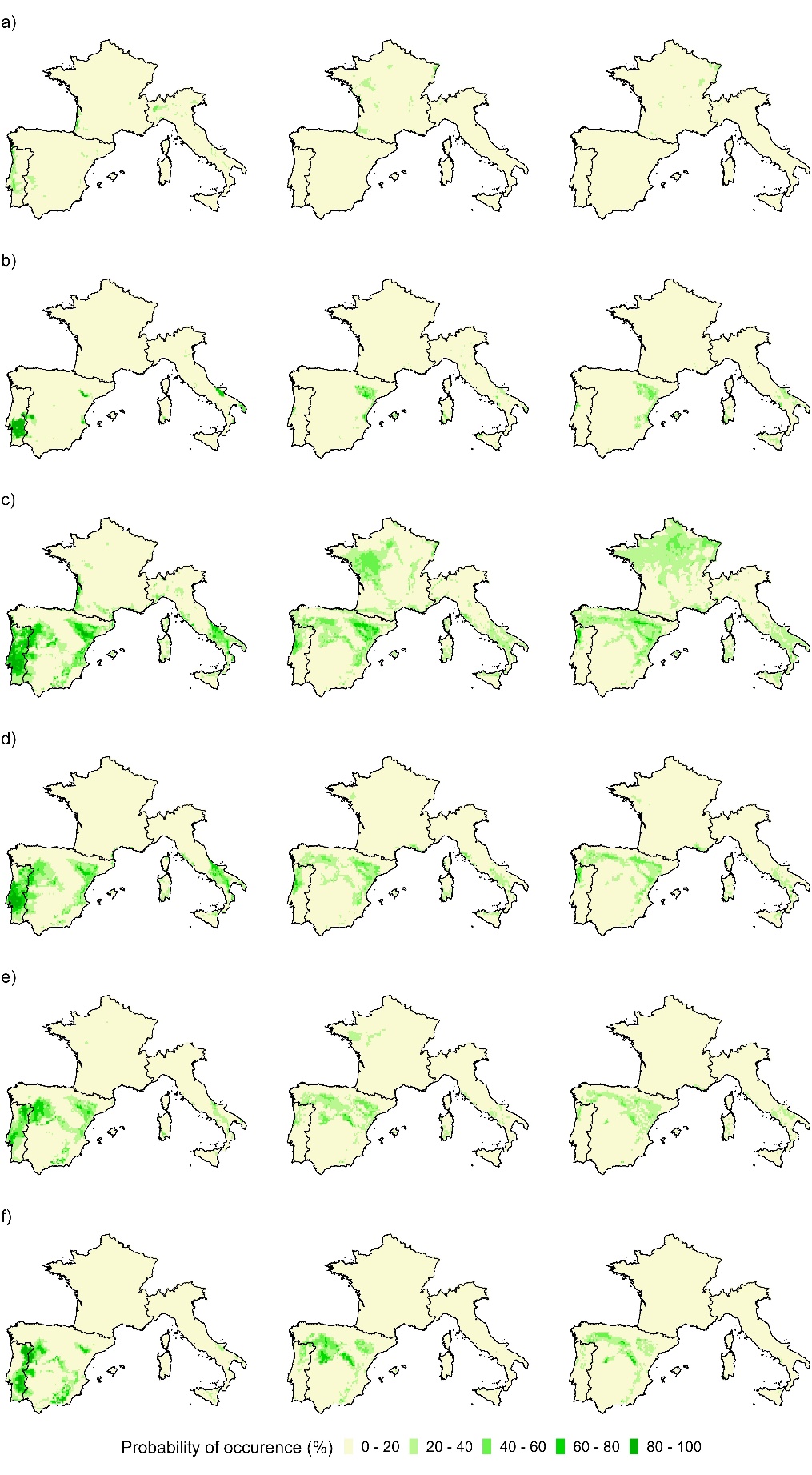


**Figure S5** – Ensemble model projected bioclimatic suitability for white varieties a) Alvarinho, b) Antão-Vaz, c) Arinto, d) Fernão-Pires, e) Malvasia-Fina, and f) Síria. Left panels: Recent past distribution of bioclimatic suitability (1989–2005). Middle panels: Future bioclimatic suitability projections (2021–2050), under the RCP 4.5 climatic scenario. Right panels: Future bioclimatic suitability projections (2051–2080), under the RCP 8.5 climatic scenario.


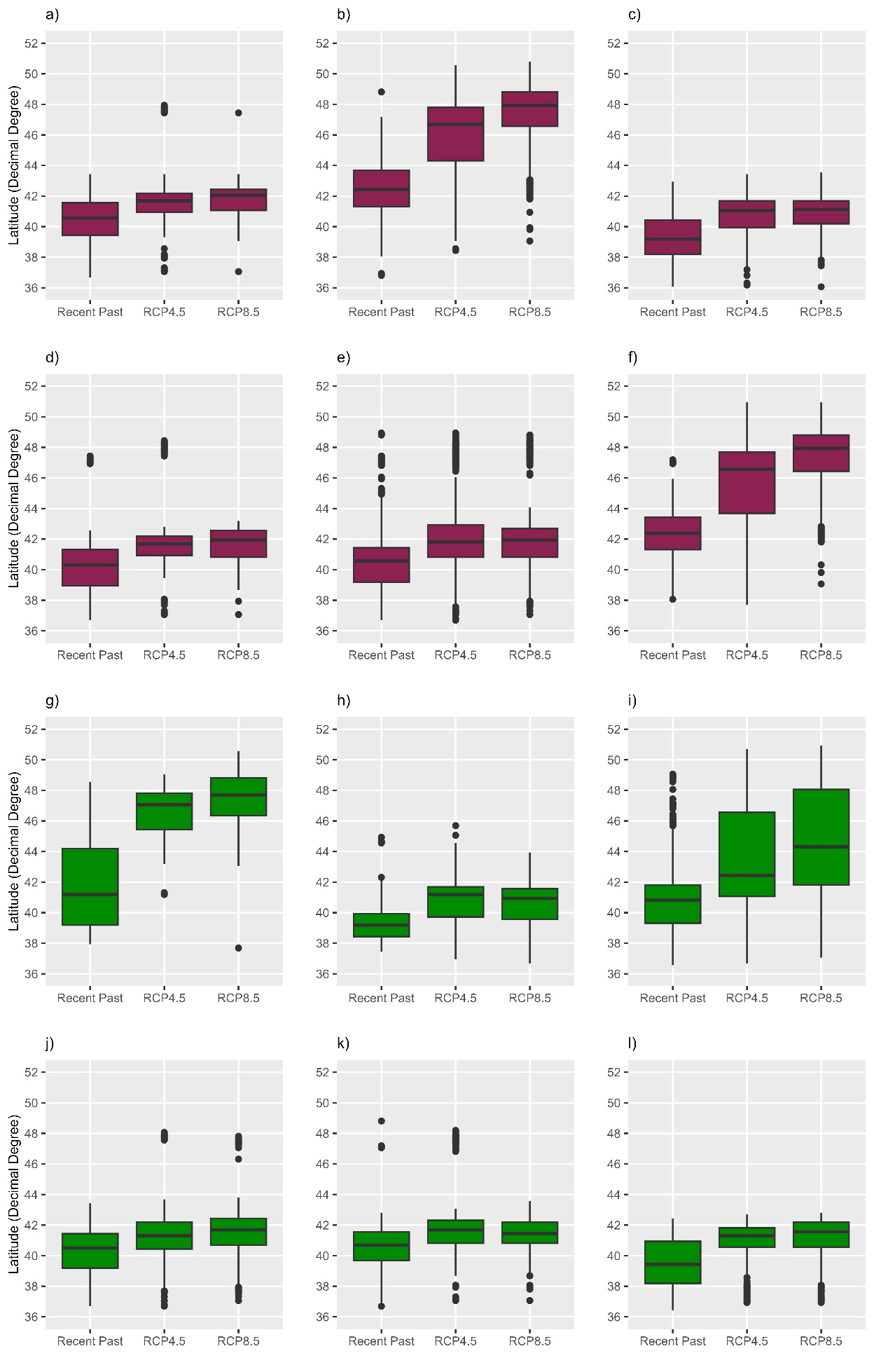


**Figure S6** – Boxplots of the distribution of bioclimatic suitability, above 20% probability of occurrence, in regards to latitude, for varieties a) Bastardo, b) Borraçal, c) Castelão, d) Touriga-Franca, e) Touriga-Nacional, f) Vinhão, g) Alvarinho, h) Antão-Vaz, i) Arinto, j) Fernão-Pires, k) Malvasia-Fina, and l) Síria, in the recent past (1989–2005) and future (2051–2080) climates. Red boxplots identify red varieties and green boxplots identify white varieties.


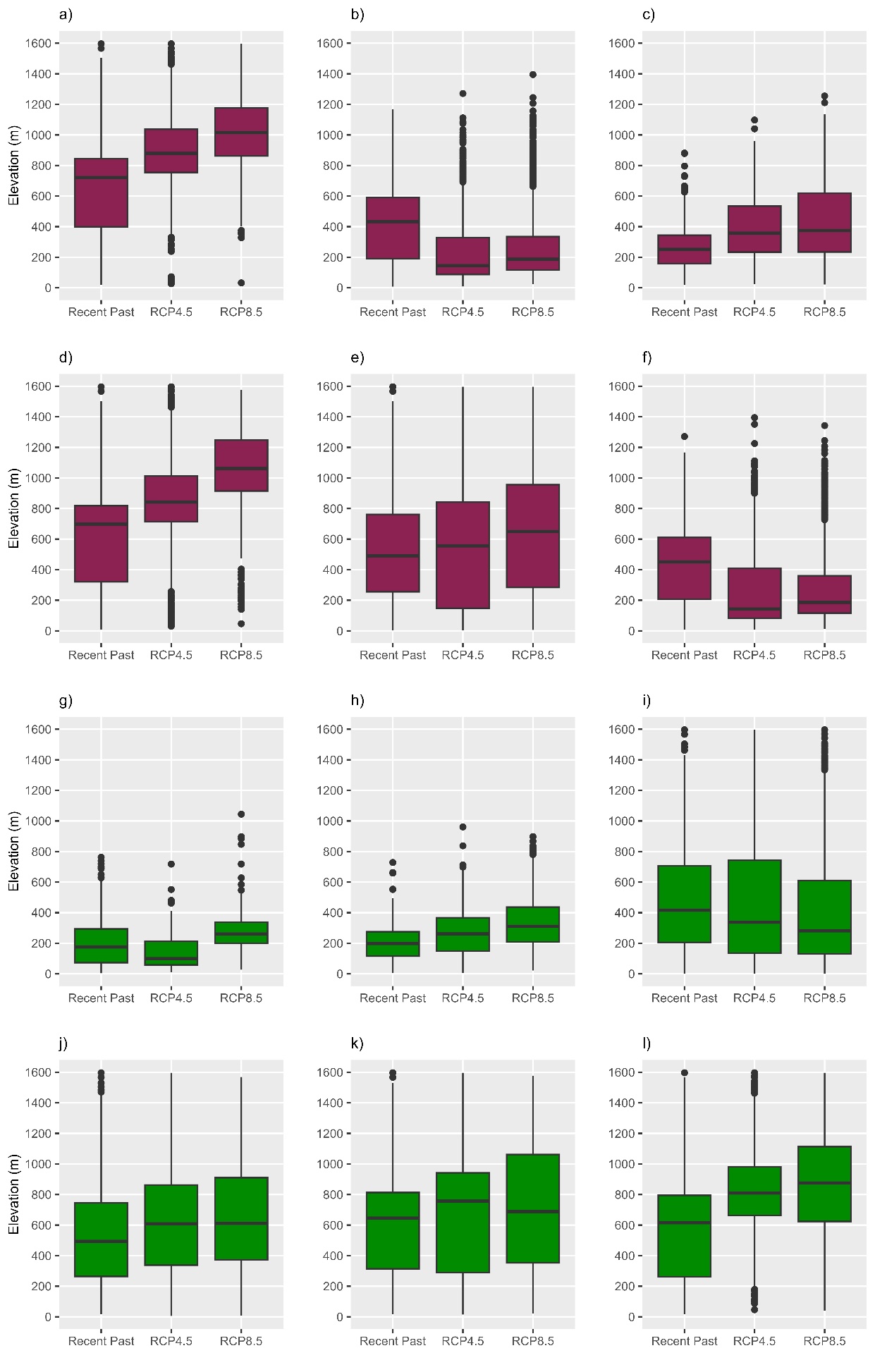


**Figure S7** – Boxplots of the distribution of bioclimatic suitability, above 20% probability of occurrence, in regards to elevation, for varieties a) Bastardo, b) Borraçal, c) Castelão, d) Touriga-Franca, e) Touriga-Nacional, f) Vinhão, g) Alvarinho, h) Antão-Vaz, i) Arinto, j) Fernão-Pires, k) Malvasia-Fina, and l) Síria, in the recent past (1989–2005) and future (2051–2080) climates. Red boxplots identify red varieties and green boxplots identify white varieties.

**Table S1** – Pearson correlation coefficients and Variance Inflation Factor scores for chosen bioclimatic indices.

|  | Pearson Correlation Coefficients | | | | Variance Inflation Factor |
| --- | --- | --- | --- | --- | --- |
| - | **Cool Night Index** | **Growing Season Precipitation** | **Huglin Index** | **Temperature Range During Ripening** | - |
| Cool Night Index | 1.000 | -0.660 | 0.940 | 0.030 | 27.200 |
| Growing Season Precipitation | -0.660 | 1.000 | -0.690 | -0.410 | 2.620 |
| Huglin Index | 0.940 | -0.690 | 1.000 | 0.320 | 25.910 |
| Temperature Range During Ripening | 0.030 | -0.410 | 0.320 | 1.000 | 4.160 |

**Table S2** - Index permutation mean scores for each grape variety.

| White Varieties | CN | GSP | HI | TRR | Red Varieties | CN | GSP | HI | TRR |
| --- | --- | --- | --- | --- | --- | --- | --- | --- | --- |
| Alvarinho | 0.562 | 0.417 | 0.950 | 0.757 | **Bastardo** | 0.407 | 0.781 | 0.114 | 0.097 |
| Antão-Vaz | 0.076 | 0.366 | 0.704 | 0.270 | **Borraçal** | 0.313 | 0.177 | 0.553 | 0.169 |
| Arinto | 0.203 | 0.630 | 0.521 | 0.218 | **Castelão** | 0.136 | 0.483 | 0.571 | 0.201 |
| Fernão-Pires | 0.170 | 0.813 | 0.388 | 0.233 | **Touriga-Franca** | 0.453 | 0.983 | 0.141 | 0.128 |
| Malvasia-Fina | 0.395 | 0.915 | 0.337 | 0.155 | **Touriga-Nacional** | 0.228 | 0.805 | 0.350 | 0.190 |
| Síria | 0.231 | 0.880 | 0.189 | 0.231 | **Vinhão** | 0.318 | 0.232 | 0.494 | 0.159 |

**Table S3** – Mean metric scores for each grape variety correlative models.

| **Alvarinho** | **GBM** | **GAM** | **CTA** | **ANN** | **RF** | **Bastardo** | **GBM** | **GAM** | **CTA** | **ANN** | **RF** |
| --- | --- | --- | --- | --- | --- | --- | --- | --- | --- | --- | --- |
| **AUC** | 0.813 | 0.894 | 0.742 | 0.713 | 0.816 | **AUC** | 0.936 | 0.950 | 0.878 | 0.902 | 0.937 |
| **TSS** | 0.633 | 0.750 | 0.461 | 0.409 | 0.635 | **TSS** | 0.797 | 0.867 | 0.643 | 0.721 | 0.791 |
| **Kappa** | 0.393 | 0.495 | 0.257 | 0.241 | 0.391 | **Kappa** | 0.688 | 0.766 | 0.587 | 0.633 | 0.688 |
| **Antão-Vaz** | **GBM** | **GAM** | **CTA** | **ANN** | **RF** | **Borraçal** | **GBM** | **GAM** | **CTA** | **ANN** | **RF** |
| **AUC** | 0.989 | 0.978 | 0.960 | 0.985 | 0.990 | **AUC** | 0.952 | 0.939 | 0.839 | 0.800 | 0.959 |
| **TSS** | 0.946 | 0.948 | 0.880 | 0.966 | 0.956 | **TSS** | 0.849 | 0.841 | 0.681 | 0.592 | 0.861 |
| **Kappa** | 0.846 | 0.867 | 0.777 | 0.868 | 0.862 | **Kappa** | 0.648 | 0.663 | 0.539 | 0.446 | 0.668 |
| **Arinto** | **GBM** | **GAM** | **CTA** | **ANN** | **RF** | **Castelão** | **GBM** | **GAM** | **CTA** | **ANN** | **RF** |
| **AUC** | 0.912 | 0.929 | 0.864 | 0.876 | 0.925 | **AUC** | 0.968 | 0.973 | 0.892 | 0.957 | 0.973 |
| **TSS** | 0.723 | 0.752 | 0.689 | 0.672 | 0.734 | **TSS** | 0.866 | 0.900 | 0.749 | 0.865 | 0.875 |
| **Kappa** | 0.657 | 0.657 | 0.631 | 0.594 | 0.676 | **Kappa** | 0.818 | 0.857 | 0.726 | 0.815 | 0.835 |
| **Fernão-Pires** | **GBM** | **GAM** | **CTA** | **ANN** | **RF** | **Touriga-Franca** | **GBM** | **GAM** | **CTA** | **ANN** | **RF** |
| **AUC** | 0.941 | 0.947 | 0.872 | 0.885 | 0.941 | **AUC** | 0.937 | 0.943 | 0.843 | 0.872 | 0.933 |
| **TSS** | 0.804 | 0.812 | 0.685 | 0.718 | 0.789 | **TSS** | 0.810 | 0.852 | 0.610 | 0.610 | 0.796 |
| **Kappa** | 0.739 | 0.765 | 0.633 | 0.606 | 0.735 | **Kappa** | 0.651 | 0.628 | 0.499 | 0.499 | 0.632 |
| **Malvasia-Fina** | **GBM** | **GAM** | **CTA** | **ANN** | **RF** | **Touriga-Nacional** | **GBM** | **GAM** | **CTA** | **ANN** | **RF** |
| **AUC** | 0.942 | 0.919 | 0.840 | 0.887 | 0.937 | **AUC** | 0.926 | 0.925 | 0.869 | 0.869 | 0.932 |
| **TSS** | 0.794 | 0.757 | 0.650 | 0.698 | 0.771 | **TSS** | 0.762 | 0.788 | 0.682 | 0.668 | 0.766 |
| **Kappa** | 0.715 | 0.655 | 0.579 | 0.575 | 0.704 | **Kappa** | 0.714 | 0.747 | 0.630 | 0.601 | 0.733 |
| **Síria** | **GBM** | **GAM** | **CTA** | **ANN** | **RF** | **Vinhão** | **GBM** | **GAM** | **CTA** | **ANN** | **RF** |
| **AUC** | 0.966 | 0.965 | 0.893 | 0.935 | 0.965 | **AUC** | 0.938 | 0.892 | 0.804 | 0.801 | 0.925 |
| **TSS** | 0.851 | 0.882 | 0.776 | 0.810 | 0.849 | **TSS** | 0.779 | 0.731 | 0.586 | 0.591 | 0.774 |
| **Kappa** | 0.789 | 0.787 | 0.698 | 0.701 | 0.782 | **Kappa** | 0.621 | 0.588 | 0.456 | 0.399 | 0.624 |
